# Supplementary material for: Diagnostic Accuracy of Artificial Intelligence in Predicting Anti-VEGF Treatment Response in Diabetic Macular Edema: A Systematic Review and Meta-Analysis
Source: J Clin Med. 2025 Nov 18;14(22):8177. doi: 10.3390/jcm14228177 (PMC12653470; doi:10.3390/jcm14228177)
Supplement: Supplementary file 1 [file jcm-14-08177-s001.zip › jcm-3891160-Tables S1-S3.pdf]

	Domain 2: Predictors	Domain 3: Outcome
atients).<br>34,495	**Low Concern**<br><br>Standard OCT images assessed consistently before outcome determination.	**Unclear Concern**<br><br>Anatomical outcome (CMT reduction) appropriate, but specific threshold and timepoint not reported, hindering reproducibility.
djustment	**Low Concern**<br><br>Standard clinical, lab, and OCT data as predictors, assessed before outcome determination.	**High Concern**<br><br>Outcome (VA prognosis) defined by data-driven median split. This dichotomization can lead to optimistic estimates and poor generalizability.
-world	**Low Concern**<br><br>Baseline OCT scan as predictor, defined and acquired consistently before outcome.	**Low Concern**<br><br>Outcome (CST reduction ≥10µm) clearly defined with justified threshold based on measurement repeatability. Appropriate 1-month timepoint.
	**Low Concern**<br><br>Predictors (OCT + clinical data) collected at baseline under trial protocol.	**Low Concern**<br><br>Composite outcome (≥5 letters VA gain AND >50µm CMT reduction) clinically relevant and clearly defined. Double-masked assessment.
lment	**Low Concern**<br><br>Standard OCT images as predictors, assessed prior to outcome.	**Low Concern**<br><br>Outcome (CST/fluid reduction improvement) clearly defined at standard timepoints (1 and 3 months).
patient<br>lear.	**Low Concern**<br><br>OCT-derived radiomic features are quantitative and consistently extracted.	**Low Concern**<br><br>Outcome (residual/recurrent DME) well-defined with specific stratified CST reduction thresholds based on established criteria.
ection bias.	**Low Concern**<br><br>Predictors (OCT, fundus photos) collected under standardized RCT protocol.	**Low Concern**<br><br>Outcome (residual fluid/HE) assessed by two blinded specialists with third adjudicator, ensuring low bias.
usceptible	**Low Concern**<br><br>Standard OCT images as predictors.	**Low Concern**<br><br>Outcome is quantitative fluid volume calculation, which is objective.
scribed,	**Low Concern**<br><br>Standard OCT and clinical data collected before outcome measurement.	**Low Concern**<br><br>Regression task (predicting exact CST/VA values) is objective. Appropriate 3-month timepoint.
ospective	**Low Concern**<br><br>OCT radiomics extracted from pre-treatment images. ROI	**Low Concern**<br><br>Outcome (Persistent DME) based on clear pre-defined criteria from

	Rating	Downgrade/Upgrade	Justification
	—	—	Comprehensive search across multiple dat
	—	—	17 retrospective cohorts, 1 RCT, 1 cross-s
	—	—	Studies with extractable 2×2 contingency
	—	—	Independent validation cohorts reported
	Moderate	**-1**	**Justification:**
	1 study (6%)		Single RCT with adequate randomization
	11 studies (61%)		Retrospective design with clear methodol
	6 studies (33%)		Selection bias, inadequate control groups
	**Serious limitations**		**Predominance of observational studies**
	Low	**0**	**Justification:**
	Direct		DME patients requiring anti-VEGF therap
	Direct		AI prediction models for treatment respon
	Direct		Relevant response definitions
	Direct		Ophthalmology clinics and research cente
	**No concerns**		**Evidence directly applicable**
	Moderate	**-1**	**Justification:**
	I^2 = 45.2% (sensitivity)		Moderate heterogeneity between studies
	I^2 = 36.1% (specificity)		Acceptable level of heterogeneity
	Explained by study quality		Meta-regression explains 78.4% of varian
y	Different AI approaches		Various algorithms and validation method
	**Some concerns**		**Moderate heterogeneity partially explai**
	Low	**0**	**Justification:**
	427 participants (meta-analysis)		Adequate for pooled estimates
	3,107 participants (total)		Large overall evidence base
	Sensitivity: 82.2-90.6%		Reasonably precise estimates
	Specificity: 71.4-83.9%		Adequate precision for decisions
	Large effect demonstrated		Significant differences
	**No concerns**		**Sufficient precision for decisions**
	Moderate	**-1**	**Justification:**
	P = 0.045		Statistically significant asymmetry
	P = 0.280		No significant rank correlation
	P = 0.156		No significant bias (modified test)
	15 studies required		Moderate significance

elation in Included Studies.

of Analysis	Patients (n)	Eyes (n)	Both Eyes Included?	Statistical Adjustment for Clustering	In An
es	104	120	Unclear	Not reported	No
	683	1,293	✓ Yes	None reported; both eyes included without adjustment	No
	53	73	Unclear	Not reported	**Ye**
nts	181	181	✗ No	Not applicable (one eye per patient)	**Ye**
nts	72	72	✗ No	Not applicable (one eye per patient)	No
	131	234	✓ Yes	None reported; both eyes included without adjustment	No
	327	327	Unclear	Not reported	**Ye**
es	12	14	✓ Yes	None reported; both eyes included without adjustment	No
	272	272	✓ Yes	None reported; both eyes included without adjustment	No
	82	113	✓ Yes	None reported; both eyes included without adjustment	**Ye**
	279	279	Unclear	Not reported	No
nts	101	101	✗ No	Not applicable (one eye per patient)	No
	281	281	✓ Yes	None reported; both eyes included without adjustment	No
es	117	133	✓ Yes	None reported; both eyes included without adjustment	No
	363	363	Unclear	Not reported	No
nts	712	712	Unclear	Not reported	**Ye**
ects	127	127	Unclear	Not reported	**Ye**
	570	570	✗ No	Not applicable (one eye per patient, post-hoc RCT)	No

n coefficient; ***RCT*** *= randomized controlled trial; — = not applicable or unable to calculate.*
