# Supplementary figures and images for: Diagnostic Accuracy of Artificial Intelligence in Predicting Anti-VEGF Treatment Response in Diabetic Macular Edema: A Systematic Review and Meta-Analysis
Source: J Clin Med. 2025 Nov 18;14(22):8177. doi: 10.3390/jcm14228177 (PMC12653470; doi:10.3390/jcm14228177)

Meta-Regression Model for Sources of Heterogeneity in Diagnostic Accuracy

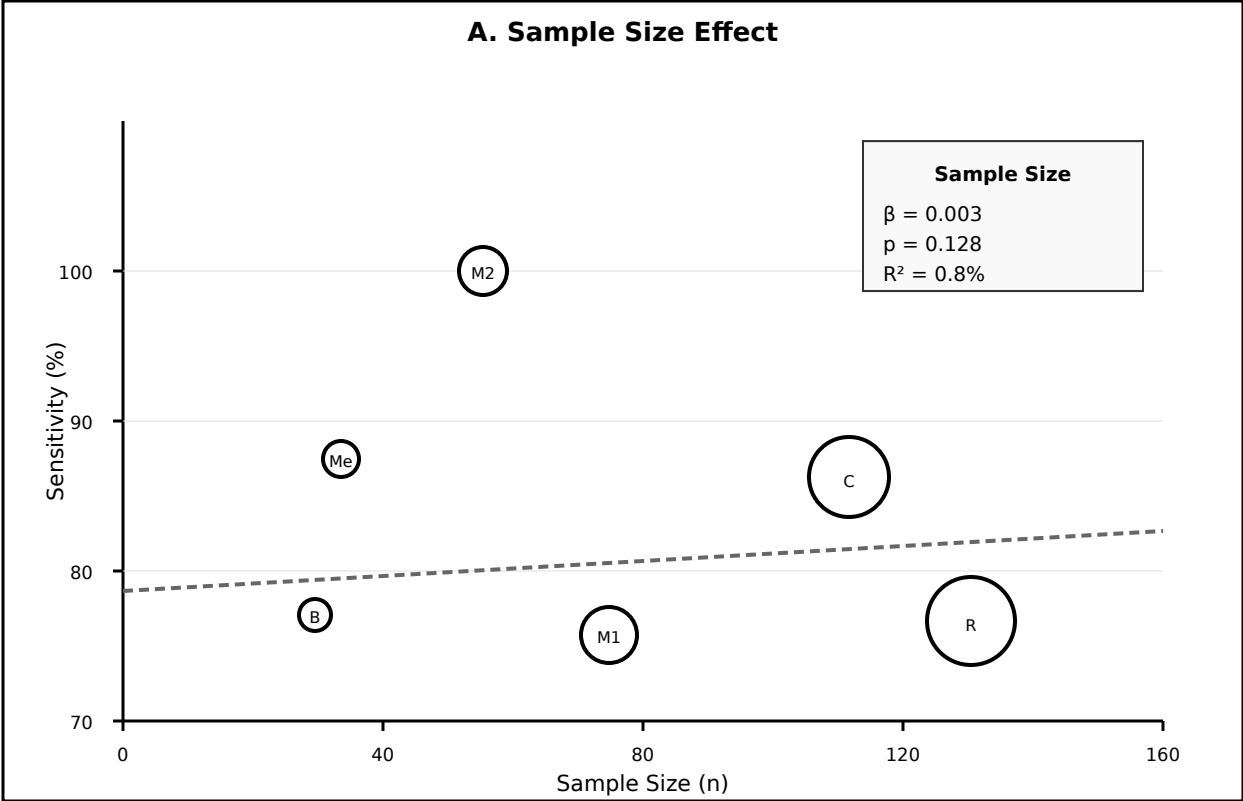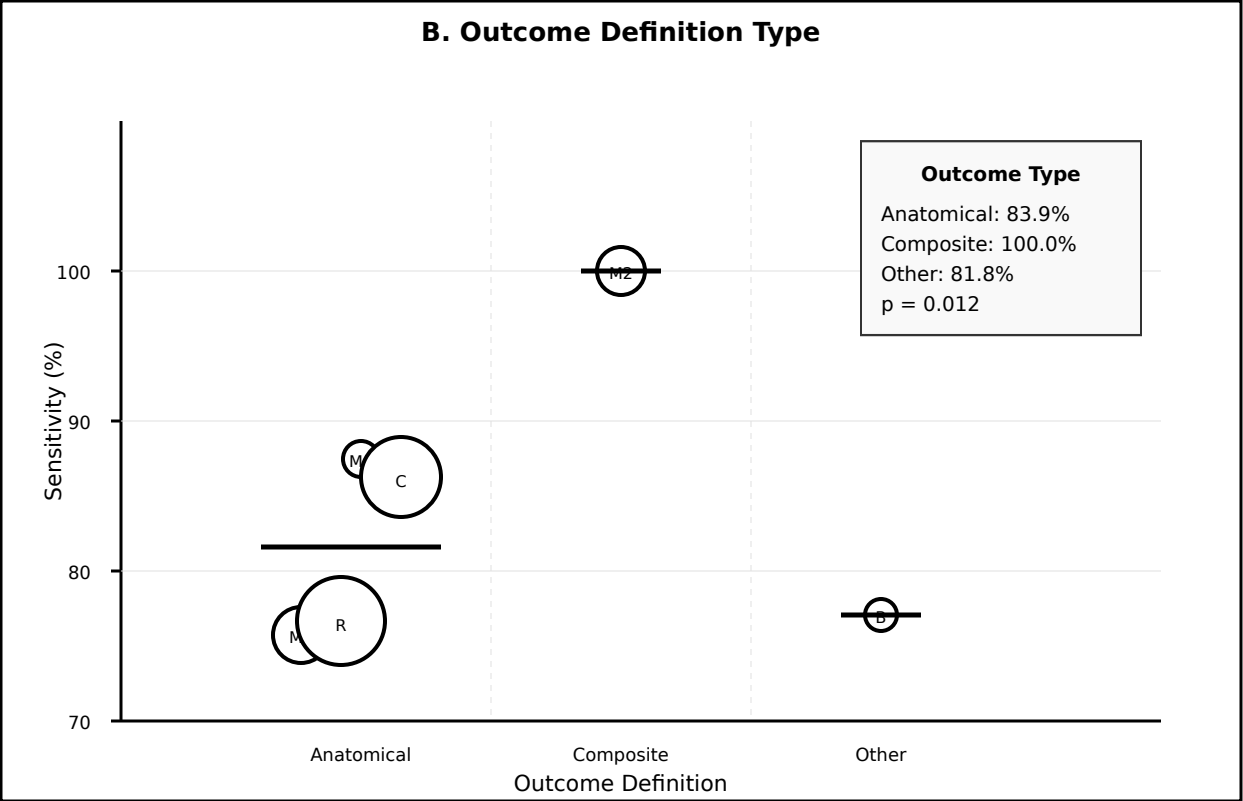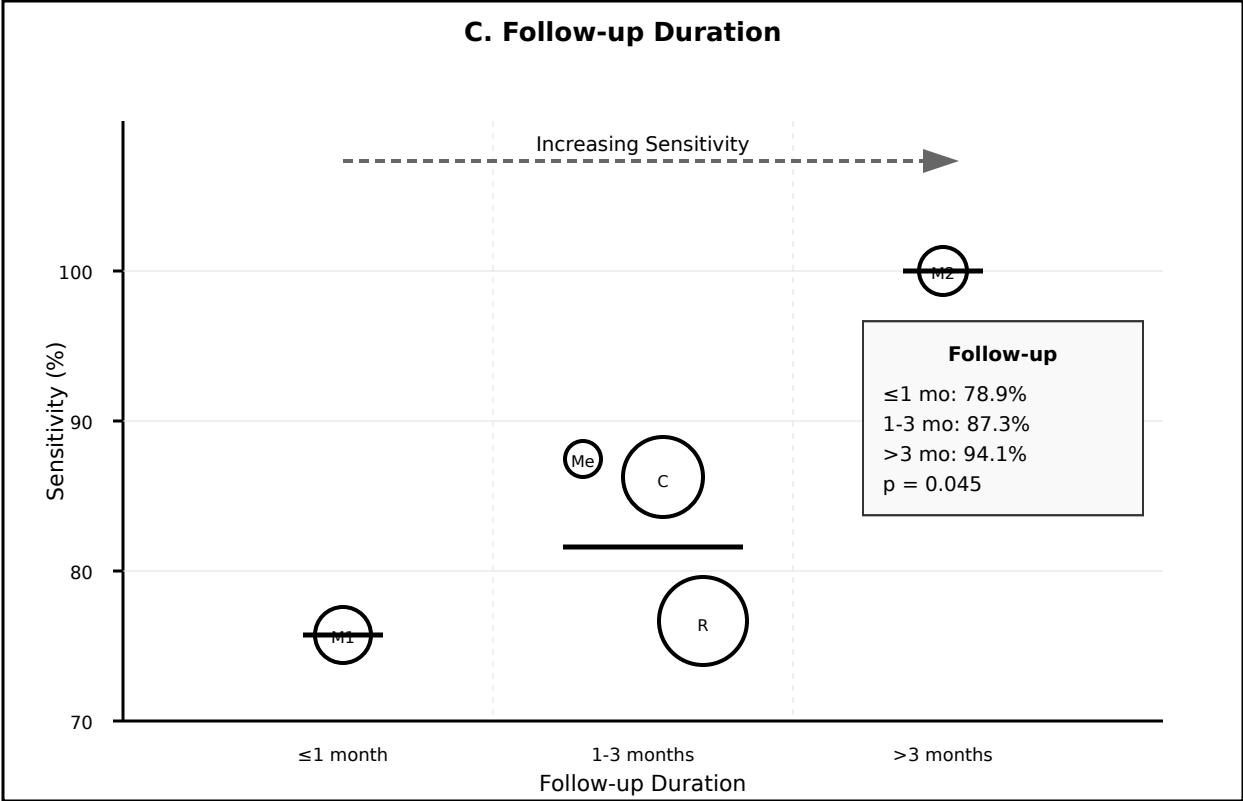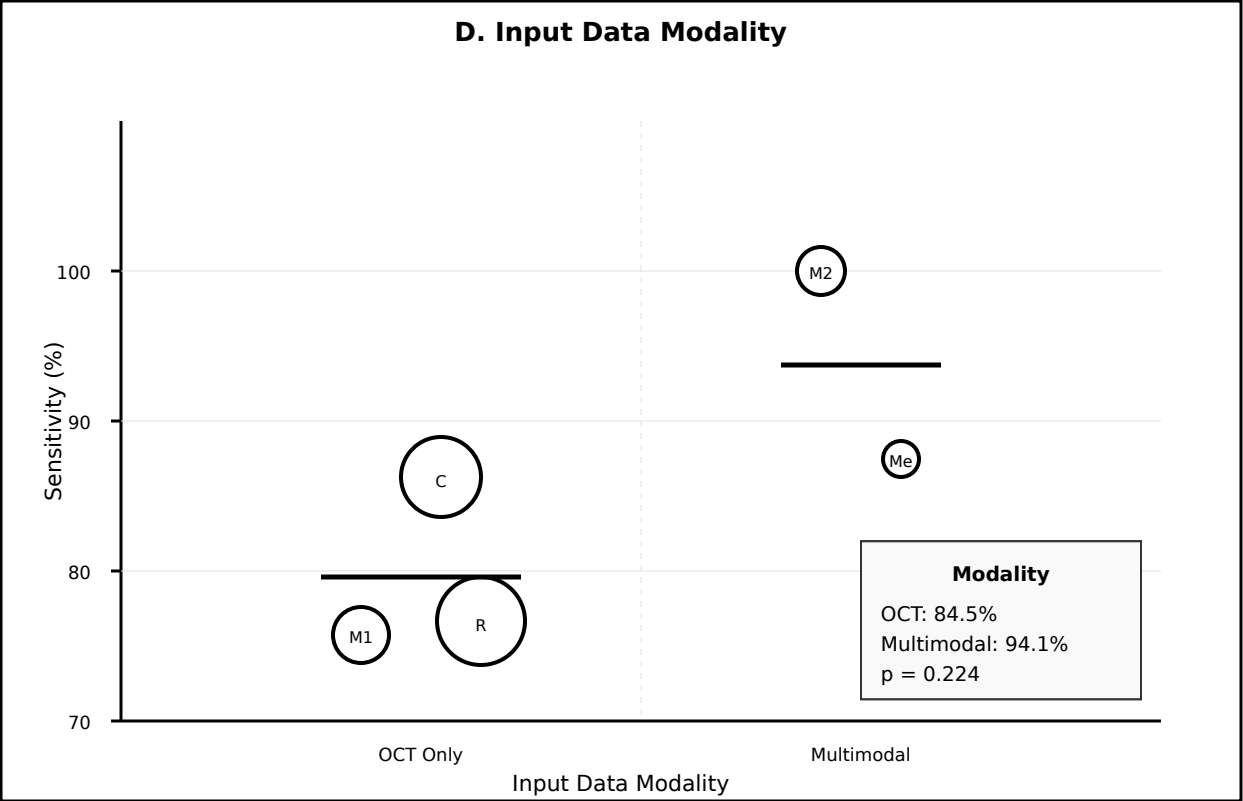

Supplement: Supplementary file 1 [file jcm-14-08177-s001.zip › Supplementary Figure S1.pdf]
